# Simulation of Antral Conditions for Estimating Drug Apparent Equilibrium Solubility after a High-Calorie, High-Fat Meal

**DOI:** 10.1021/acs.molpharmaceut.4c01038

**Published:** 2025-01-15

**Authors:** Christos Reppas, Christina Chorianopoulou, Ioanna Karkaletsi, Shirin Dietrich, Andriani Bakolia, Maria Vertzoni

**Affiliations:** Department of Pharmacy, National and Kapodistrian University of Athens, Zografou 15771, Greece

**Keywords:** equilibrium solubility, fed state, gastric aspirates, FEDGAS, FeSSGF-V3

## Abstract

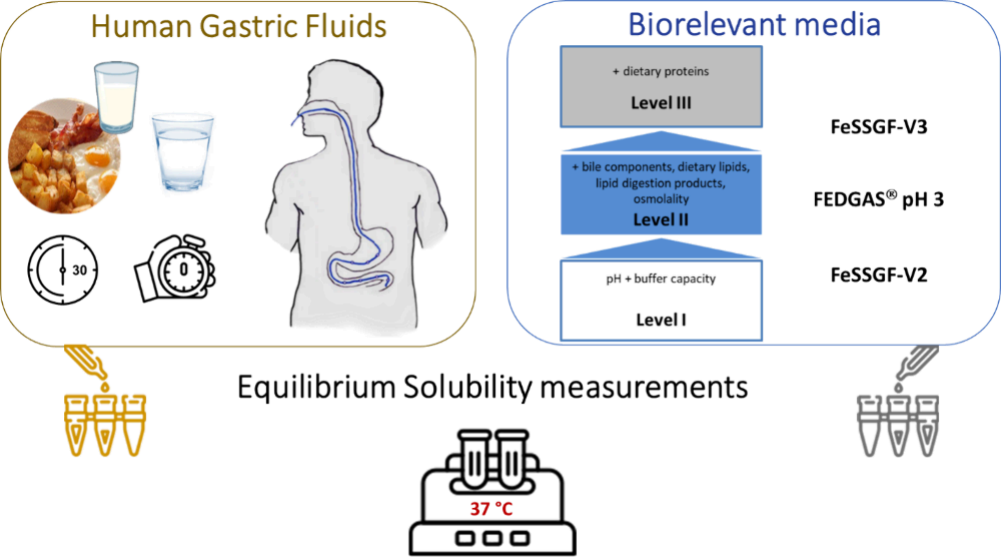

The simulation of antral conditions for estimating drug apparent equilibrium solubility after a high-calorie, high-fat meal is challenging. In this study, (1) we measured the apparent equilibrium solubility of two model lipophilic drugs, ketoconazole and danazol, in antral aspirates collected at various time points after a minced high-calorie, high-fat meal and a glass of water 30 min after initiation of meal administration, and we designated one point estimate for ketoconazole and one point estimate for danazol; (2) we evaluated the usefulness of FeSSGF-V2 and FEDGAS pH = 3 in reproducing the two point estimates; (3) we evaluated potential compositions of FeSSGF-V3 that simulate the pH, the buffer capacity toward both less acidic and more acidic values, and the antral lipid and protein contents with easily accessible, commercially available products, and (4) we identified the most useful composition of FeSSGF-V3 for reproducing the two point estimates. For both model drugs, apparent solubility in FeSSGF-V2 and in FEDGAS pH 3 deviated substantially from the corresponding point estimate. For FeSSGF-V3, hydrochloric acid, acetates, and FEDGASbuffer pH 3 were evaluated for regulating the pH and buffer capacity, FEDGASgel was used for simulating the lipid content, and Régilait skimmed milk powder was used for simulating the protein content. Level III FeSSGF-V3 prepared with hydrochloric acid, 6.1% (w/v) Régilait, and 2.83% (w/v) FEDGASgel, i.e., one-sixth of FEDGASgel concentration in FEDGAS pH 3, was comparatively the most useful medium for point estimating ketoconazole and danazol apparent solubility in antral contents after water administration in the fed state, induced as requested by regulatory authorities in oral drug bioavailability studies. Level III FeSSGF-V3 prepared by using hydrochloric acid as the principal pH controlling species could be useful in the evaluation of food effects on drug absorption with *in silico* physiologically based biopharmaceutics modeling approaches and, also, with biorelevant in vitro methodologies.

## 1 Introduction

Depending on the type of administered dosage form, drug apparent equilibrium solubility in the contents of the gastrointestinal lumen affects the rates at which the drug becomes available in the dissolved state in the luminal contents and/or reflects the capacity of luminal contents to solubilize the drug.

Based on an analysis performed by scientists from the United States Food and Drug Administration back in 2018, physiologically based pharmacokinetic (PBPK) models together with a clinical pharmacokinetic profile under fasted state conditions described ≈50% (23/48) of the observed food effects on total exposure within a predefined boundary of 25%.^1^ In a more recent analysis performed by the International Consortium for Innovation and Quality in Pharmaceutical Development (IQ consortium), it was concluded that, in cases where food effects were predicted with low confidence, PBPK-based food effect predictions could be improved if, among others, food effects on intragastric drug apparent solubility are better estimated,^2^ in situations where the drug empties primarily from the antrum and not via the stomach road.

As a mechanistic understanding of the drivers of drug solubility in the antral contents after a high-calorie, high-fat meal and their integration into PBPK models is not possible today, the use of *in vitro* estimated physiologically relevant value(s) as input parameter(s) to a PBPK model could increase reliability.

About 15 years ago, it was proposed to estimate intragastric drug apparent solubility in the fed state based on data collected in media simulating the intragastric environment at various time periods, after administration of a liquid meal with composition, origin of calories, calorie content, and volume similar to that of a high-calorie, high-fat meal.^3,4^ One issue with those proposals is that the collection of relevant data is limited by various analytical challenges. The use of a single fed state simulated gastric fluid (FeSSGF)^3,5^ and consideration of the level of simulation of the intragastric environment required for the evaluation of a specific drug or drug product^5^ have been proposed for avoiding unnecessary sample preparation efforts in certain situations. However, relevant solubility data are still rarely generated during drug discovery and/or drug development programs and the use of commercially available products is considered key for increasing the easiness of relevant solubility measurements.^2^

On the other hand, it has been shown that the physicochemical characteristics of antral contents including the pH gradient over time after a high-calorie, high-fat meal and a glass of water 30 min after initiation of meal administration^6−9^ deviate from those after a liquid meal with composition, origin of calories, calorie content, and volume similar to those of a high-calorie, high-fat meal.^10^ As a result, FeSSGF-V2 has been proposed to replace FeSSGF.^8^

The present study had three objectives.

The first was to measure ketoconazole and danazol apparent equilibrium solubility in recently collected antral aspirates at various time points after a minced high-calorie, high-fat meal and a glass of water 30 min after initiation of meal administration to healthy young adults,^9^ and to designate point estimates for the apparent solubility of ketoconazole and danazol. Ketoconazole is a lipophilic compound (clogP 4.4, intrinsic aqueous solubility ≈2 μg/mL) with a strong pH-dependent solubility due to its specific weakly basic characteristics [p*K*_a1_ 2.94 and p*K*_a2_ 6.51 (both basic)] [e.g., refs (4,11)]. Danazol is a poorly soluble, nonionizable lipophilic compound [(logP 4.53, aqueous solubility in buffer solution (pH 7.5) 0.8 μg/mL].^12^

The second objective was to evaluate the usefulness of two recently proposed media, FeSSGF-V2^8^ and FEDGAS pH = 3,^13^ in reproducing the point estimates for the apparent equilibrium solubility of ketoconazole and danazol in antral contents after a high-calorie, high-fat meal and a glass of water 30 min after initiation of meal administration to healthy young adults.

The third objective was to identify the most appropriate principal pH controlling species, and the required level of biorelevance^5^ of a new medium, FeSSGF-V3, for obtaining the best estimate of ketoconazole and of danazol apparent equilibrium solubility in the antral contents after a high-calorie, high-fat meal and a glass of water 30 min after initiation of meal administration to healthy young adults.

## 2 Materials and Methods

### 2.1 Materials

Ketoconazole was supplied by Janssen (Beerse, Belgium). Danazol (USP, micronized powder) was manufactured by Coral Drugs Pvt. Limited (Murthal, India). Acetic acid ≥99.8%, was from Fluka Honeywell (Muskegon, USA). Hydrochloric acid, concentrated 37%, was from Sigma-Aldrich (Saint Louis, USA). Sodium chloride, analytical grade purity, was from Merck KGaA (Darmstadt, Germany). Sodium hydroxide, ≥99.8%, was from Fluka Honeywell (Muskegon, USA). Lipofundin MCT/LCT 20% was from B. Braun Melsungen AG (Melsungen, Germany). FEDGAS High-Fat Meal Late (pH = 3) Dissolution Kit (buffer concentrate and gel) was from Biorelevant, Ltd. (London, United Kingdom). Régilait Skimmed Milk Powder [36% proteins, 52% carbohydrates, 0.8% fats, 1.1% salt (w/w), https://www.regilait.com/en/products/powdered-milks/instant-skimmed-milk-powder-0-fat/] (Burgundy, France) was purchased locally.

### 2.2 Physicochemical Characterization of Solubility Media

Buffer capacity was measured by titrating 1 mL of the sample with HCl solution (0.1 M) and 1 mL with NaOH solution (0.1 M) until the pH of the sample is modified by one unit.

Osmolality was measured based on the depression of the sample’s freezing point [osmometer, model Semi Micrο Typ Dig. L, KNAUER GmbH (Germany)].

Apparent viscosity was measured at 37 °C with a rotational viscometer (RM 100 CP 2000 Plus, Lamy Rheology, France) equipped with a measuring cone and plate system MK-CP4005. A sample volume of 146 μL was placed on the viscometer’s plate, and the measuring cone was lowered in measuring position. The software Visco-RM Soft software was used to control the instrument. Each measurement was performed at ascending shear rates of 50, 100, and 200 s^–1^. The duration for each step was set at 10 s.

### 2.3 Human Gastric Fluids Used in Solubility Measurements

Human gastric fluids (HGF_fed_) were aspirated from the antrum of healthy young adults, at various times after a minced high-calorie, high-fat meal {toasted bread, eggs, bacon, fried potatoes, whole milk, and butter (989 kcal, 61% fat), ≈530 mL, 423 mOsmol/kg^6^} and 240 mL water 30 min after initiation of meal administration in a recently performed clinical study.^9^ Inclusion criteria, exclusion criteria, and demographic characteristics of the subjects participated in the clinical study have been published.^9^ Single doses of paracetamol and itraconazole had been administered in the clinical study.^9^ However, paracetamol is nonionized under the gastric conditions and itraconazole was present in very low apparent concentrations during the aspiration period ranging between LLOQ (<0.060 μg/mL) and 7.9 μg/mL;^14^ therefore, paracetamol and itraconazole presence in aspirated samples is unlikely to affect the pH of antral contents, due to the high buffer capacity of antral contents.^9^

In the clinical study, the amount and composition of the meal and the administration of water after it reflect the conditions applied in bioavailability/bioequivalence studies worldwide (e.g., (15,16)). Unlike other types of meals, e.g., rice pudding meal or roast chicken and vegetable meal, which produce a very heterogeneous intragastric distribution of solid and liquid,^17^ heterogeneity of gastric contents 30 min after initiation of the specific high-calorie, high-fat meal is reduced.^18,19^ Also, it has been shown that the apparent viscosity is only slightly affected after mincing, primarily by decreasing its variability,^6^ likely due to the decreased presence of >2 mm particles (from 25% of meal volume to 10% of meal volume) and the increased presence of 0.1–1 mm particles (from 21% of meal volume to 38% of meal volume) with most of meal particles not affected by meal mincing (54% of meal volume).^6^

Upon their aspiration, individual HGF_fed_ samples were maintained at −70 °C, until used in this study. On the day of solubility experiment, aspirated samples^9^ from a total of seven volunteers were brought to room temperature and pooled. Pooling was performed exclusively on a volume availability basis.

On the day of solubility measurement, individual aspirates collected at 45, 75, 105, and 135 min were pooled to create four HGF_fed_ samples. HGF_fed_^45^ was created by mixing 2 mL individual aspirates collected after 10 administrations to six volunteers and thus forming a pooled sample of 20 mL. HGF_fed_^75^ was created by mixing 1 mL individual aspirates collected after 10 administrations to six volunteers and, thus, forming a pooled sample of 10 mL. HGF_fed_^105^ was created by mixing 2 mL individual aspirates collected after 11 administrations to seven volunteers and, thus, forming a pooled sample of 22 mL. HGF_fed_^135^ was created by mixing 2 mL individual aspirates after 11 administrations to seven volunteers and, thus, forming a pooled sample of 22 mL. The pH, buffer capacity, and osmolality of pooled samples measured immediately before solubility measurements are presented in Table 1. Due to limited volume availability, pH, buffer capacity, and osmolality of pooled samples were measured only once. However, based on previously collected data in pooled antral aspirates from a similarly performed clinical study,^6,8^ variability of pH, buffer capacity, and osmolality measurements in pooled samples generated after one freeze–thaw cycle is expected to be low (Supporting Information, SI1). pH, buffer capacity, and osmolality of pooled samples used in the present investigation were generally similar to the median values estimated from individual data collected previously after 16 single administrations to eight volunteers^8,9^ (Table 1); only buffer capacity of the HGF_fed_^135^ pooled sample seems to deviate from the average values estimated immediately upon aspirations (Table 1), suggesting also that the use of aspirates from six (not seven) volunteers for the creation of HGF_fed_^45^ and HGF_fed_^75^ pooled samples does not introduce significant bias in the discussion of apparent solubility data.

**Table 1:** Physicochemical Characteristics of Pooled Antral Aspirates Collected in a Recently Performed Clinical Study^9^ and Used in the Present Investigation (*n* = 1) *vs.* the Median [Range] of Individual Data Collected Previously after 16 Single Administrations to Eight Volunteers in Two Separate Clinical Studies^8,9^^a^

		HGF_fed_^45^	median [range] of individual data at 45 min	HGF_fed_^75^	median [range] of individual data at 75 min	HGF_fed_^105^	median [range] of individual data at 105 min	HGF_fed_^135^	median [range] of individual data at 135 min
pH		4.4	4.5 [2.8–5.5][9]	4.0	4.0 [1.9–5.6][9]	3.8	3.7 [1.7–5.6] [9]	3.6	3.5 [1.7–4.9] [9]
buffer capacity [(mmol/L)/ΔpH]	titration with HCl	16.0	19 [6.5–42] [9]	19.2	24 [12–144] [9]	20.0	22 [5.1–200][9]	19.6	26.4 [8.6–390] [9]
	titration with NaOH	12.0	12.5 [4.0–20.0] [9]	14.0	18.7 [8.0–25.0] [9]	18.0	18.5 [4.5–24.0] [9]	15.2	18.4 [6.4–29.8] [9]
osmolality (mOsmol/kg)		192	ΝΜ^a^	238	290 [90–402] [8]	274	ΝΜ^a^	296	307 [94–447] [8]

a ΝM = not measured.

### 2.4 Biorelevant Media Used in Solubility Measurements

Biorelevant media were prepared on the day of the solubility measurements.

#### 2.4.1 Level II and Level I FeSSGF-V2

Composition and physicochemical characteristics of Level II FeSSGF-V2 are presented in Table 2. Mean(SD) (*n* = 3) osmolality of Level II FeSSGF-V2 was measured to be 293.0(1.7) mOsmol/kg, in line with the average osmolality of the aqueous phase of antral contents 75 and 135 min after a glass of water administered 30 min post initiation of meal intake (Table 1).

**Table 2:** Composition and Physicochemical Characteristics of Level I and Level II FeSSGF-V2

		Level I	**Level II**
sodium oleate (mM)^a^			0.044
phosphatidylcholine (mM)^a^			0.674
triglycerides (mM)^a^			13.7
sodium chloride (mM)			127.5
*ortho*-phosphoric acid (mM)		5.5	5.5
sodium dihydrogen phosphate (mM)		32	32
HCl/NaOH		qs pH 3	qs pH 3
		
osmolality (mOsmol/kg)			293.0(1.7)^b^
buffer capacity [(mmol/L)/ΔpH]	titration with HCl	24.83(0.76)^b^	24.83(0.76)^b^
	titration with NaOH	3.6(0.0)^b^	3.6(0.0)^b^
pH		3.0	3.0

a By using Lipofundin, 4.5% v/v.

b Mean(SD) (*n* = 3) based on measurements performed in this study.

Level I FeSSGF-V2 differs from Level II FeSSGF-V2 in that it does not contain NaCl or Lipofundin (Table 2). Mean(SD) (*n* = 3) buffer capacity of Level I FeSSGF-V2 measured with HCl solution was 23.67(0.58) mmol/L/ΔpH, in line with the overall median buffer capacity of antral contents measured with HCl solution (24.0 mmol/L/ΔpH (*n* = 71),^9^). In contrast, the buffer capacity of Level I FeSSGF-V2 measured with NaOH solution was 3.53(0.06) mmol/L/ΔpH, much lower than the overall median buffer capacity of antral contents measured with NaOH solution (18.0 mmol/L/ΔpH (*n* = 76),^9^).

#### 2.4.2 FEDGAS pH = 3, FEDGAS_1/2_ pH = 3, FEDGAS_1/4_ pH = 3, and FEDGASbuffer pH = 3

The composition and the physicochemical characteristics of FEDGAS pH = 3 (Level II biorelevance^5^) is presented in Table 3.

**Table 3:** Composition and Physicochemical Characteristics of FEDGAS at pH = 3

FEDGAS gel^a^ (g/90 mL FEDGAS)		15.3
FEDGAS buffer concentrate^a^ (g/90 mL FEDGAS)		3.7
osmolality (mOsmol/kg)		505(15)^b^
buffer capacity [(mmol/L)/ΔpH]	titration with HCl	25.67(0.58)^b^
	titration with NaOH	25.17(0.76)^b^
pH		3.0

a Citrates, included in the FEDGAS High-Fat Meal Late (pH = 3) Dissolution Kit (biorelevant.com).

b Mean(SD) (*n* = 3) based on measurements performed in this study.

In contrast with Level II FeSSGF-V2, the osmolality of FEDGAS pH = 3 (Level II biorelevance^5^), i.e., 1.7–3.3 times the mean osmolality values of the aqueous phase of antral contents estimated at various times between 45 and 225 min after administration of a high-calorie, high-fat meal and a glass of water to healthy fasted adults.^8^

FEDGA*S*_1/2_ pH = 3 (Level II biorelevance^5^) was prepared as FEDGAS pH = 3 but with half the amount of FEDGASgel, i.e., by using 7.65 g of FEDGASgel/90 mL. Mean(SD) (*n* = 3) osmolality of FEDGA*S*_1/2_ pH = 3 was measured to be 271.7(1.5) mOsmol/kg, i.e., 0.9–1.8 times the mean osmolality values of the aqueous phase of antral contents estimated at various times between 45 and 225 min after administration of a high-calorie, high-fat meal and a glass of water to healthy fasted adults.^8^

FEDGAS_1/4_ pH = 3 (Level II biorelevance^5^) was prepared as FEDGAS pH = 3 but with 1/4 of the amount of FEDGASgel, i.e., by using 3.825 g of FEDGASgel/90 mL. Mean(SD) (*n* = 3) osmolality of FEDGAS_1/4_ pH = 3 was measured to be 168.67(0.58) mOsmol/kg, i.e., 0.5–1.1 times the mean osmolality values of the aqueous phase of antral contents estimated at various times between 45 and 225 min after administration of a high-calorie, high-fat meal and a glass of water to healthy fasted adults.^8^

FEDGASbuffer pH = 3 (Level I biorelevance^5^) was prepared by diluting FEDGAS buffer concentrate with distilled water, as indicated in Table 3. Unlike with Level I FeSSGF-V2, mean(SD) (*n* = 3) buffer capacity of FEDGASbuffer pH = 3 (FEDGASbuffer) measured with NaOH was 21.00(0.00) mmol/L/ΔpH, and when measured with HCl, it was 24.0(1.7) mmol/L/ΔpH, in line with the overall median buffer capacity of antral contents measured with NaOH solution (18.0 mmol/L/ΔpH) and measured with HCl solution (24.0 mmol/L/ΔpH).^9^

#### 2.4.3 Level III, Level II, and Level I FeSSGF-V3

In Level III FeSSGF-V3, the presence of proteins was simulated using Régilait Skimmed Milk Powder. The high-calorie, high-fat meal consists of up to about 1000 kcal and ≈15% of the total amount of calories is derived from proteins.^15,16^ If 1 g of protein corresponds to 4 kcal, then the meal contains up to about 37 g of proteins. Based on phenol red data, after a high-calorie, high-fat meal and a glass of water 30 min after initiation of meal intake, the impact of resting gastric contents/swallowed saliva/gastric secretions on the dilution of antral contents is substantial; however, dilution of contents occurs predominantly during meal administration.^8^ Specifically, when 100 mg of phenol red was included in the meal, the mean phenol red concentration in the antrum fluctuated around 0.06 mg/mL for at least 3 h after initiation of meal administration.^6^ It can, therefore, be assumed that the protein concentration in the antral region would be about 37 g × (0.06 mg/mL/100 mg) = 0.0222 g/mL ≈ 22 mg/mL. Since Régilait contains 36% w/w proteins, Régilait Skimmed Milk Powder 6.1% w/v in medium is equivalent to 2.196% (w/v) protein concentration, i.e., ≈ 22 mg/mL.

Level II FeSSGF-V3 differs from Level III FeSSGF-V3 in that it does not contain Régilait Skimmed Milk Powder but it contains sodium chloride (Table 4). In both Level II FeSSGF-V3 and Level III FeSSGF-V3, FEDGASgel corresponds to 16.6% (1/6) of the amount that is present in FEDGAS pH = 3 [2.83% w/v (Table 4) vs 16.6% w/v (15.3 g/90 mL, Table 3)]; osmolality was measured to be in line with the osmolality of the aqueous phase of antral contents 105, 165, and 225 min after a high-calorie, high-fat meal and a glass of water at 30 min after initiation of meal administration (mean values were 259, 304, and 260 mOsmol/kg, respectively^8^).

**Table 4:** Composition and Physicochemical Characteristics of Level I, Level II, and Level III FeSSGF-V3 Prepared when Using Hydrochloric Acid, Acetates, or FEDGASbuffer for Simulating the Antral pH and Buffering Capacity^a^

		**FeSSGF-V3**_**HCl**_			**FeSSGF-V3**_**Acetates**_			**FeSSGF-V3**_**FEDGASbuffer**_		
		Level I	**Level II**	**Level III**	Level I	**Level II**	**Level III**	Level I	**Level II**	**Level III**
**Régilait (% w/v)**^b^				6.1			6.1			6.1
**FEDGAS gel (% w/v)**^c^			2.83	2.83		2.83	2.83		2.83	2.83
**sodium chloride (mM)**			137.2			55.9			82.5	
**hydrochloric acid (mM)**		1.2	2.0	65.0						
**acetic acid (mM)**					135.0	135.0	67.5			
**FEDGAS buffer concentrate**^d^ (% v/v)								4.08	4.08	1.0
**sodium hydroxide (mM)**					2.0	2.0	0.75	8.5	8.5	1.5
**apparent viscosity at 100 s**^**–1**^**(mPa·s)**				207 (52)			248 (18)			386 (20)
**osmolality (mOsmol/kg)**			316.0 (1.1)	329.5 (1.8)		308.0 (1.5)	368.3 (1.5)		303.5 (1.0)	310.0 (1.0)
**buffer capacity [(mmol/L)/ΔpH]**	**measured with HCl**	10.55 (0.84)	10.55 (0.84)	24.5 (1.0)	17.60 (0.20)	17.60 (0.20)	27.50 (0.50)	35.33 (0)	35.33 (0)	28.33 (0.14)
	**measured with NaOH**	1.133 (0.058)	1.133 (0.058)	15.33 (0.29)	20.66 (0)	20.66 (0)	27.70 (0.29)	26.22 (0.19)	26.22 (0.19)	23.75 (0.25)
**pH**		3.0	3.0	3.0	3.0	3.0	3.0	3.0	3.0	3.0

a Apparent viscosity, osmolality, and buffer capacity were measured in triplicate and data are presented as Mean(SD).

b Equivalent protein content of 22 mg/mL.

c 16.6% of the amount that is currently recommended (www.biorelevant.com).

d Citrates, included in the FEDGAS High-fat Meal Late (pH = 3) Dissolution Kit (biorelevant.com).

Level I FeSSGF-V3 differs from Level III FeSSGF-V3 in that it does not contain Régilait Skimmed Milk Powder, FEDGASgel, or sodium chloride (Table 4). Buffer capacity of Level I FeSSGF-V3 is closer to intragastric data only when acetates are used as buffering species; HCl has minimal resistance to increasing the pH (i.e., it has very low buffer capacity) when titrating with NaOH (1.1 mmol/L/ΔpH, Table 4) and it has lower resistance in decreasing the pH compared with antral contents (10.6 mmol/L/ΔpH (Table 4) vs 24.0 mmol/L/ΔpH^9^). FEDGASbuffer shows higher resistance to decreasing pH compared with antral contents (35.3 mmol/L/ΔpH (Table 4) vs 24.0 mmol/L/ΔpH^9^). The presence of Régilait Skimmed Milk Powder Level III FeSSGF-V3 impacts buffer capacity substantially, making Level III FeSSGF-V3_HCl_ comparatively the most appropriate for simulating the intragastric environment regarding the pH, buffer capacity, osmolality, lipid content, protein content, and apparent viscosity (Table 4vs. refs (8,9,14)).

Details on the method of preparation of Level I, Level II, and Level III FeSSGF-V3 media proposed and tested in the present investigation are provided in the Supporting Information, SI2.

In Level III FeSSGF-V3 media, within-day and between-day variability of pH, buffer capacity, and osmolality were low; the coefficient of variation was less than 4.4% for pH and buffer capacity and less than 9.4% for osmolality (Supporting Information, SI3). Within-day and between-day variability of the apparent viscosity of Level III FeSSGF-V3 biorelevant media at 100 s^–^^1^ and at 37 °C was higher; the coefficient of variation was 20–25% (Supporting Information, SI3). Also, storage at room temperature for 1 h or shaking at 37 °C for 24 h does not impact the pH or buffer capacity; coefficients of variation were similar to the within-day and between-day coefficients of variation of freshly prepared media (Supporting Information, SI3). Storage at room temperature for 1 h does not impact the apparent viscosity at 100 s^–1^. Shaking at 37 °C for various time periods up to 24 h decreases the apparent viscosity at 100 s^–1^ by 16–32% at 24 h (Supporting Information, SI3).

### 2.5 Solubility Measurements

Apparent solubility of ketoconazole and of danazol was measured with the shake-flask method at 37 °C and 75 opm using a shaking and thermostatic water bath [Unitronic Laboratory Water Bath, J.P. Selecta SA (Arbera, Spain)]. Preliminary experiments with ketoconazole and danazol in Level III FeSSGF-V3_HCl_ and in the HGF_fed_^15^ sample created from aspirates collected previously^6^ after one freeze–thaw cycle indicated that equilibrium is achieved within 4 h of incubation. Relevant data are presented as the Supporting Information (SI4).

For each measurement, 2 mL was transferred into an amber glass vial containing the drug substance in excess (3 mg for danazol and 30 mg for ketoconazole) and 2 mL was transferred into an amber glass vial containing no drug (blank sample). Solubility measurements were performed in triplicate, whereas one blank sample was evaluated. The four vials were sealed tightly and placed in a shaking water bath for 4 h. At equilibrium, the pH of the blank sample (pH_eq_) remained unchanged, in all cases, i.e., any deviation of pH_eq_ from the initial pH is not related to instability/change in composition of the medium. When using Level I of Level II media, at equilibrium, each sample was filtered through a regenerated cellulose filter (regenerated cellulose membranes 17 mm, pore diameter 0.45 μm Titan3 Thermo Fisher Scientific, Waltham, Massachusetts, USA). Level III media and HGF_fed_ samples could not be not filtered. At equilibrium, separation of the undissolved drug was achieved by centrifuging at 11,400*g* at 37 °C for 10 min. In 300 μL of the supernatant, 600 μL of acetonitrile was added. Then, the sample was centrifuged for 10 min at 11,400*g* at 10 °C and the clear supernatant, after appropriate dilution with mobile phase, was injected to the HPLC system.

Based on experiments performed earlier with the same filters used in the present investigation, ketoconazole shows negligible adsorption from aqueous buffers with pH ranging from 3.5 and 5^4^ (data are presented in Supporting Information, SI5). Also, danazol shows negligible adsorption from Level II FeSSIF^20^ (data are presented in Supporting Information, SI5). Adequacy of centrifugation vs filtration for ketoconazole and danazol has been confirmed by using media, which could also be filtered, i.e., canine intestinal fluids for ketoconazole^21^ and Level II FeSSIF for danazol,^20^ and the same filters that were used in the present investigation.

Due to volume availability limitations, the numbers of replications, *n*, of ketoconazole solubility measurements in HGF_fed_^75^ and HGF_fed_^135^ samples were *n* = 1 (as implied also by the small volume of the HGF_fed_^75^ sample, Section 2.3) and *n* = 2 (due to breaking of one of the three vials), respectively.

### 2.6 Drug Assay Methods

Ketoconazole and danazol concentrations in biorelevant media and in antral aspirates were determined using previously validated HPLC-UV methods.^22,23^ In all cases, quantification was based on standard curves constructed by using the corresponding medium. LOQ and LOD were 60 and 20 ng/mL for both ketoconazole and danazol, respectively.

### 2.7 Data Analysis

Depending on the normality test and the equal variance test, one–way analysis of variance or the Kruskal–Wallis test at the 0.05 level of significance using SigmaPlot for Windows Version 11.0 (Systat Software, Inc., San Jose, California, USA) was applied to estimate the impact of time after water administration in the fed state on ketoconazole and on danazol apparent equilibrium solubility in antral contents.

## 3 Results

### 3.1 Ketoconazole and Danazol Apparent Equilibrium Solubility in Pooled HGF_fed_ Samples and Point Estimates

**Figure 1:**
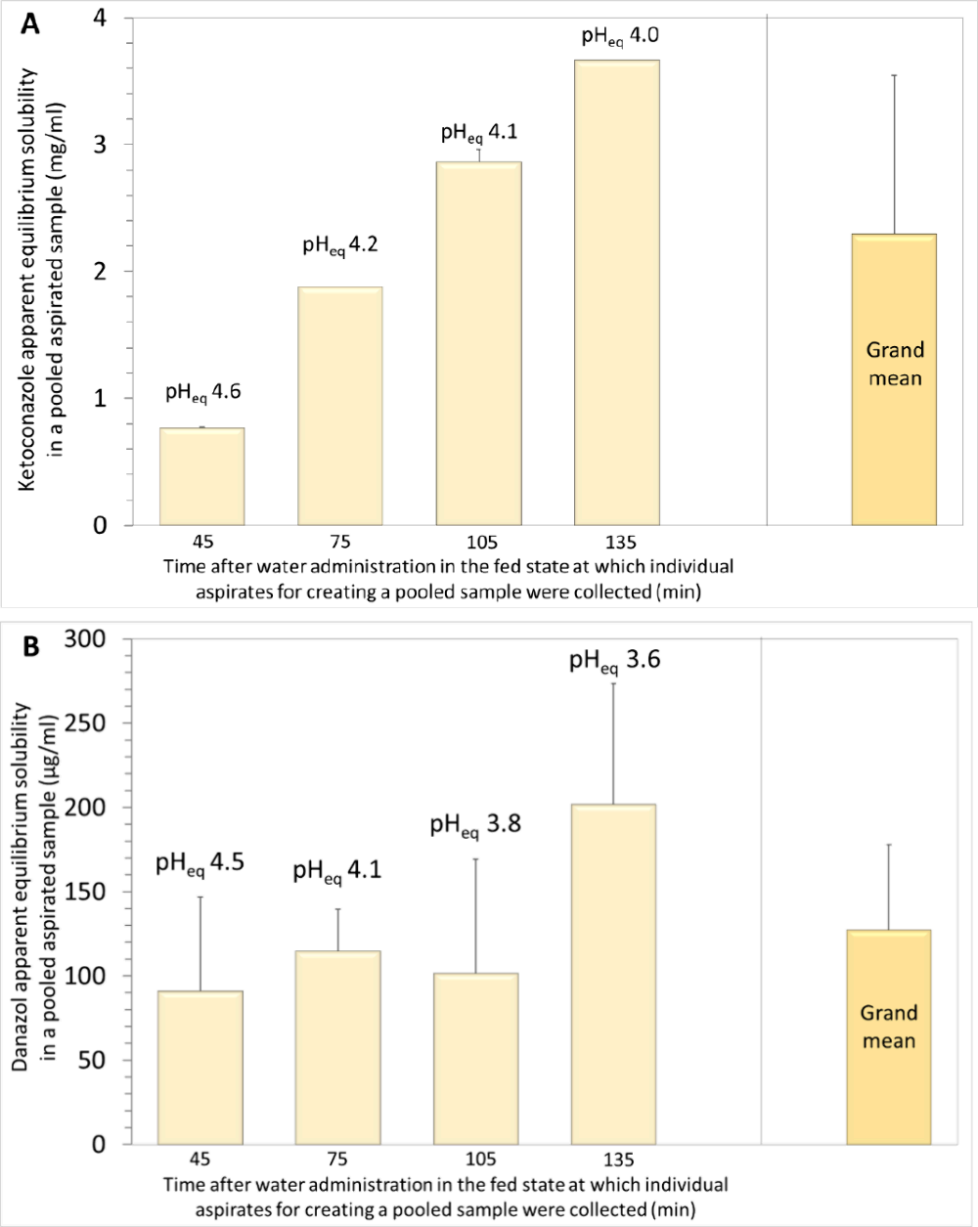
Apparent equilibrium solubility of ketoconazole (A) and danazol (B) in four pooled aspirates created from individual aspirates collected from the antrum of healthy young adults 45, 75, 105, and 135 min after water administration in the fed state, induced as requested by regulatory authorities in oral drug bioavailability studies (Section 2.3) and the calculated grand mean+SD (*n* = 4). pH_eq_ is the pH at equilibrium. Mean+SD (*n* = 3) data are presented in all cases, except for ketoconazole data in HGF_fed_^75^ (*n* = 1) and in HGF_fed_^135^ (*n* = 2) for which SD values are not shown.

Ketoconazole apparent solubility data in pooled HGF_fed_ samples are presented in Figure 1A. pH_eq_ values (Figure 1Α) were 0.2–0.4 units higher than the initial pH values of the HGF_fed_ samples (Table 1). Although the normality test passed, i.e., the mean value could be used as an estimate of apparent solubility in each HGF_fed_ sample, the equal variance test failed. Based on the result of the Kruskal–Wallis test, differences in the apparent solubility among the four HGF_fed_ samples are greater than would be expected by chance, i.e., they are statistically significant (*p* = 0.005). However, perhaps due to the small number of replications and the lower power of distribution-free tests, post hoc testing for identifying differences between two HGF_fed_ samples did not indicate any statistically significant difference.

Mean apparent equilibrium solubility in pooled aspirates ranged from 0.77(0.01) to 3.67(0.04) mg/mL (Figure 1A). It may be worth mentioning that ketoconazole apparent solubilities in human antral fluids collected 30, 60, and 120 min after 500 mL of Ensure Plus (liquid, 753 kcal, 32% fat, 27% proteins, 41% carbohydrates) were 0.63 mg/mL (pH_eq_ 5.7), 3.15 mg/mL (pH_eq_ 5.1), and 4.7 mg/mL (pH_eq_ 4.9), respectively.^4^ The dosing conditions (no water administration after Ensure Plus), the fat content of Ensure Plus, and the pH values of aspirated samples after 500 mL of Ensure Plus are all different after a high-calorie, high-fat, meal, making the comparison of solubility data in antral aspirates reported by Diakidou et al.^4^ with data from this study extremely difficult.

Solubilities of ketoconazole in simple aqueous solutions were estimated (based on ref (11)) to be 0.17, 0.41, 0.52, and 0.65 mg/mL, in pH 4.6, 4.2, 4.1, and 4.0, respectively. These values indicate that the aqueous solubilities of ketoconazole at pH_eq_ observed in HGF_fed_^45^, HGF_fed_^75^, HGF_fed_^105^, and HGF_fed_^135^ samples account for 21, 22, 18, and 18%, respectively, of the measured apparent solubility in the corresponding HGF_fed_ sample. Therefore, the apparent solubility in HGF_fed_ samples is primarily due to the presence of solubilizing species. The contributions of solubilizing species to the apparent equilibrium solubility in HGF_fed_^45^, HGF_fed_^75^, HGF_fed_^105^, and HGF_fed_^135^ samples are 0.61, 1.47, 2.34, and 3.00 mg/mL, respectively (1.85 mg/mL on average).

The grand mean apparent ketoconazole solubility estimated from the four mean values in HGF_fed_^45^, HGF_fed_^75^, HGF_fed_^105^, and HGF_fed_^135^ (Figure 1A) is 2.29 mg/mL. However, in the present investigation, drug apparent solubility was measured in pooled aspirates collected after administration of a minced high-calorie, high-fat meal. Based on data collected earlier by using a 500 kcal meal, the pH in the antrum may overestimate gastric pH after chewing and swallowing of the same meal by up to one unit.^19^ Based on data collected with the Heidelberg capsule^24^ and with the SmartPill capsule,^7^ the median pH 45–105 min after initiation of chewing and swallowing a high-calorie, high-fat meal (900–1000 kcal) is about 3. Assuming pH_eq_ 3.7, the contribution of the aqueous phase to the ketoconazole apparent equilibrium solubility is 1.29 mg/mL (based on ref (11)). By taking also into account the average contribution of solubilizing species estimated from data in this study (1.85 mg/mL), a value of 3.14 mg/mL is estimated, i.e., an apparent solubility of 3 mg/mL being better than 2.29 mg/mL reflects the point estimation of ketoconazole solubility in antral contents during the first couple hours after administration of glass of water in the fed state.

Danazol apparent solubility in HGF_fed_ samples does not change significantly with the aspiration time (*p* = 0.156, power = 0.23) (Figure 1B). pH_eq_ values (Figure 1B) were practically identical to the initial pH values of HGF_fed_ samples (Section 2.3). Mean(SD) (*n* = 3) values in HGF_fed_^45^, HGF_fed_^75^, HGF_fed_^105^ were and HGF_fed_^135^, 91(56), 115(25), 102(68), and 202(72) μg/mL, respectively.

The grand mean solubility estimated from the four mean values in HGF_fed_^45^, HGF_fed_^75^, HGF_fed_^105^, and HGF_fed_^135^ is 127.3 μg/mL. Therefore, 130 μg/mL could be considered as a point estimate of danazol apparent solubility in gastric contents during the first couple hours after administration of glass of water in the fed state.

### 3.2 Usefulness of FeSSGF-V2 and FEDGAS pH = 3 in Point Estimating Ketoconazole and Danazol Apparent Equilibrium Solubility in Antral Contents

**Figure 2:**
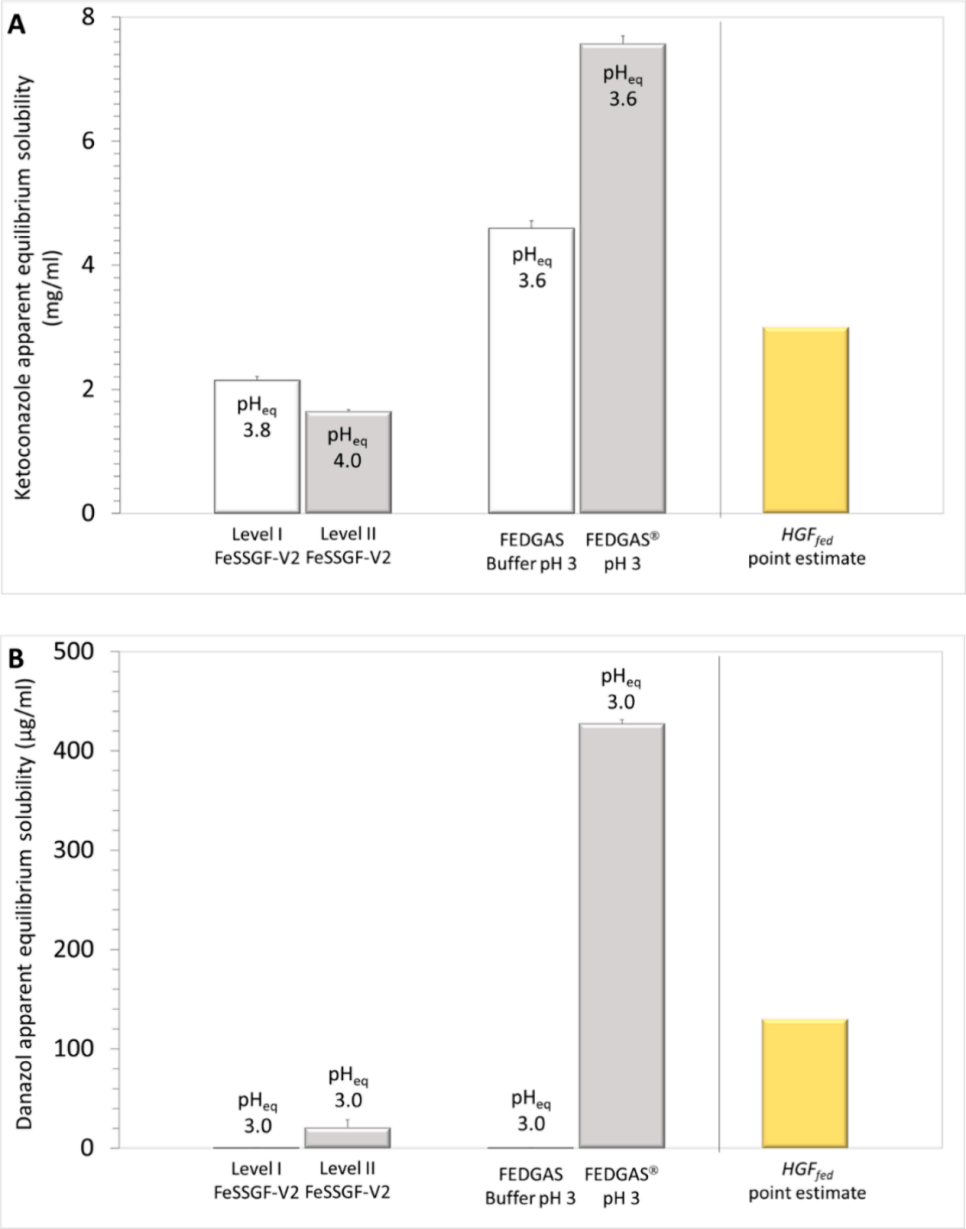
Mean+SD (*n* = 3) apparent equilibrium solubility of ketoconazole (A) and danazol (B) in Level I FeSSGF-V2 Level II FeSSGF-V2, FEDGASbuffer, and FEDGAS pH = 3 in comparison with the point estimate for the apparent drug solubility in antral contents of healthy adults, based on the corresponding grand mean for solubility in antral contents (Figure 1). pH_eq_ is the pH at equilibrium.

Ketoconazole apparent equilibrium solubility in Level I FeSSGF-V2 [2.141(0.067) mg/mL] is 30% lower than the point estimate for intragastric solubility, and in FEDGASbuffer pH 3 (Level I biorelevance) [4.60(0.12) mg/mL] 50% higher than the point estimate for intragastric solubility (HGF_fed_, 3 mg/mL, Figure 2A). It has been shown that differences in cationic counterions in buffers (potassium versus sodium) at the same buffer capacity (50 mM) by potentially affecting the final polymorph or the pH can make a difference.^25^

Ketoconazole apparent equilibrium solubility in Level II FeSSGF-V2 [1.639(0.035) mg/mL] is more than 40% lower than the point estimate for intragastric ketoconazole apparent equilibrium solubility and in FEDGAS pH = 3 (Level II biorelevance) [7.57(0.13) mg/mL] more than 2.5 times higher than the point estimate of intragastric ketoconazole apparent equilibrium solubility (Figure 2A). Ketoconazole solubility in FEDGAS_1/2_pH = 3 (Level II biorelevance) [5.653(0.020) mg/mL] and in FEDGAS_1/4_pH = 3 (Level II biorelevance) [5.07(0.19) mg/mL] (Figure 2A) is also more than 1.6 times higher than the point estimate of intragastric apparent solubility. The buffer species of FEDGASbuffer pH 3 and the lipid employed in FEDGASgel seem to be the main reasons for the overestimation of intragastric equilibrium solubility of ketoconazole by data in FEDGAS.

Danazol apparent equilibrium solubility in Level I FeSSGF-V2 [0.253(0.013) μg/mL] and in FEDGASbuffer pH = 3 (Level I biorelevance) [0.429(0.066) μg/mL] is more than two orders of magnitude lower than the point estimate of danazol apparent equilibrium solubility in antral contents (HGF_fed_, 130 μg/mL, Figure 2B).

Danazol apparent equilibrium solubility in Level II FeSSGF-V2 [20.5(7.9) μg/mL] is 80% lower than the point estimate for the intragastric apparent solubility in antral contents. In contrast, danazol apparent equilibrium solubility in FEDGAS pH = 3 (Level II biorelevance) [427.3(4.0) μg/mL] is more than three times higher than the point estimate for intragastric apparent solubility (HGF_fed_, 130 μg/mL, Figure 2B). Danazol solubility in FEDGAS_1/2_pH = 3 (Level II biorelevance) was 234.3(3.6) μg/mL, and in FEDGAS_1/4_pH = 3 (Level II biorelevance), it was 118.7(2.9) μg/mL with the latter being closest to the point estimate for danazol apparent solubility in antral contents (HGF_fed_, 130 μg/mL, Figure 2B).

### 3.3 Usefulness of FeSSGF-V3 in Point Estimating Ketoconazole and Danazol Apparent Equilibrium Solubility in Antral Contents

**Figure 3:**
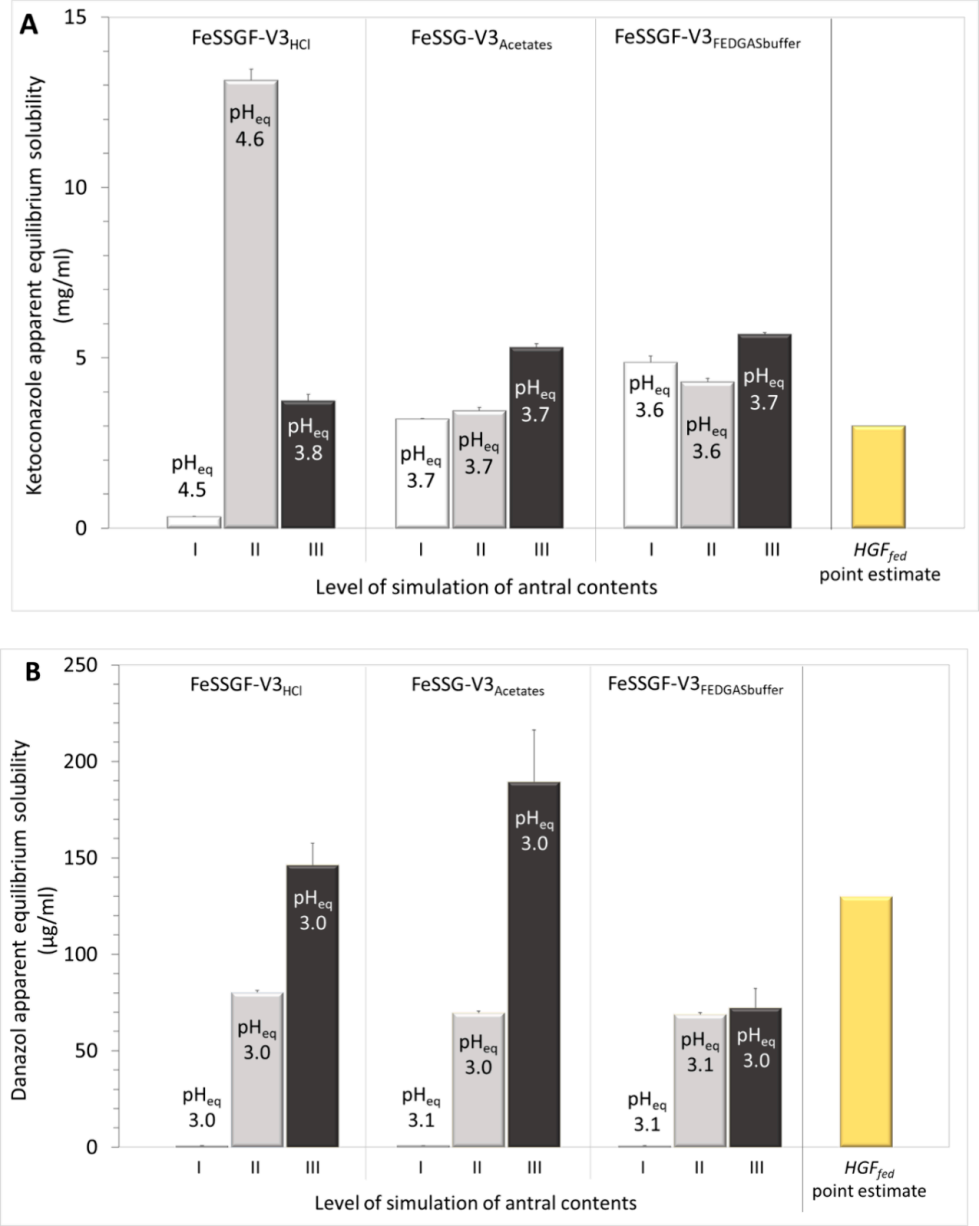
Mean+SD (*n* = 3) apparent equilibrium solubility of ketoconazole (A) and danazol (B) in FeSSGF-V3 prepared by using HCl, acetates, and FEDGASbuffer to achieve pH = 3 and reflecting the intragastric environment after water administration in the fed state at simulation Levels I, II, and III in comparison with the point estimate for the apparent drug solubility in antral contents of healthy adults, based on the corresponding grand mean for solubility in antral contents (Figure 1). pH_eq_ is the pH at equilibrium.

Ketoconazole apparent equilibrium solubility in Level I FeSSGF-V3 media varies with the buffer capacity of the medium and pH_eq_ (Figure 3A). Buffer species identity can influence equilibrium solubility of ionizable compounds. They can either raise solubility by forming more soluble compound–buffer complexes or decrease it by forming compound–buffer precipitates (e.g., salting out of bases below pH max or of acids above pH max).^25^ At Level I simulation, ketoconazole apparent solubility in FeSSGF-V3_acetates_ (3.20 mg/mL) was comparatively closest to the point estimate for ketoconazole solubility in intragastric contents (3 mg/mL, Figure 3A).

In Level II FeSSGF-V3_HCl_, lipid species greatly enhance the apparent solubility. Compared with the initial pH = 3, pH_eq_ was 4.6 > [(ketoconazole pka_1_ 2.9) + 1], i.e., at pH_eq_, the ketoconazole molecule is mostly single protonated (e.g., https://www.iq.usp.br/gutz/Curtipot_.html). The ketoconazole molecule at pH_eq_ has higher affinity with lipid species than the ketoconazole at pH 3 where half of the ketoconazole molecule is monoprotonated and half is diprotonated, leading to an apparent solubility that is four times higher than the point estimate for the intragastric apparent solubility (HGF_fed_, Figure 3A). In contrast, in Level II FeSSGF-V3_acetates_ or Level II FeSSGF = V3_FEDGAS_, pHeq < [(ketoconazole pka_1_ 2.9) + 1], the ionized fraction of ketoconazole is substantial; the apparent solubility is affected much less by the presence of lipid species, and it is similar to the apparent solubility in Level I FeSSGF-V3_acetates_ or Level I FeSSGF-V3_FEDGASbuffer_ (Figure 3A). At Level II simulation, ketoconazole apparent solubility in FeSSGF-V3_acetates_ (3.45 mg/mL) was comparatively closest to the point estimate for intragastric ketoconazole solubility (HGF_fed_, 3 mg/mL, Figure 3A).

In Level III FeSSGF-V3_HCl_, the buffering effect of proteins limits pH_eq_ to 3.8. At this pH, the presence of diprotonated ketoconazole is substantial [about 14% of ketoconazole molecules (e.g., https://www.iq.usp.br/gutz/Curtipot_.html)] and the impact of lipid species on ketoconazole apparent solubility is smaller compared with Level II FeSSGF-V3_HCl_. In Level III FeSSGF-V3_acetates_ and in Level III FeSSGF-V3_FEDGASbuffer_, proteins do not affect pH_eq_; however, they increase ketoconazole solubilization (Figure 3A). At Level III simulation, the apparent ketoconazole solubility in FeSSGF-V3_HCl_ (3.73 mg/mL) was comparatively closest to the point estimate for ketoconazole solubility in antral contents (Figure 3A).

Notably, the inclusion of physiologically relevant amounts of dietary proteins increases the apparent viscosity (Table 4) to physiologically relevant levels;^14^ after 16 administrations to eight healthy adults, the overall median apparent viscosity of aspirates collected from the antrum 75–225 min after initiation of meal administration (45–195 min after water administration) has been estimated to be 433 mPa·s at 100 s^–1^.^9^ The impact of dietary protein addition on the apparent viscosity could be attributed, at least partly, to the presence of solid particles in the medium (observed visually during the experiments in this study) and could further be related to the buffer species identity (the apparent viscosity of Level III FeSSGF-V3buffer is almost double the apparent viscosity of Level III FeSSGF-V3_HCl_, Table 4).

As expected, in all danazol solubility measurements, pH_eq_ was almost identical to the initial pH (Figure 3B).

Mean(SD) (*n* = 3) danazol apparent equilibrium solubilities in Level I FeSSGF-V3_HCl_, Level I FeSSGF-V3_acetates_, and Level I FeSSGF-V3_FEDGASbuffer_ were 0.663(0.045) μg/mL, 0.753(0.088) μg/mL, and 0.64(0.12) μg/mL, respectively, i.e., more than two orders of magnitude lower than the point estimate for danazol solubility in antral contents (HGF_fed_, 130 μg/mL, Figure 3B).

Mean(SD) (*n* = 3) danazol apparent equilibrium solubilities in Level II FeSSGF-V3_HCl_, Level II FeSSGF-V3_acetates_, and Level II FeSSGF-V3_FEDGASbuffer_ were 80.1(1.2) μg/mL, 69.67(0.82) μg/mL, and 68.8(1.0) μg/mL, respectively, i.e., at least 38% lower than the point estimate for danazol apparent equilibrium solubility in antral contents (HGF_fed_, 130 μg/mL, Figure 3B).

Mean(SD) (*n* = 3) danazol apparent equilibrium solubilities in Level III FeSSGF-V3_HCl_, Level III FeSSGF-V3_acetates_, and Level III FeSSGF-V3_FEDGASbuffer_ were 146(11) μg/mL, 190(27) μg/mL, and 72(10) μg/mL, respectively. As with ketoconazole, danazol solubility in Level III FeSSGF-V3_HCl_ was closest to the point estimate for the apparent solubility in antral contents (Figure 3A,B). It is worth mentioning that, in contrast with danazol data in Level I FeSSGF-V3 and Level II FeSSGF-V3 (Figure 3B):

1. the apparent danazol equilibrium solubility in Level III FeSSGF-V3 media varied with the identity of the species employed for regulating the buffer capacity in Level I and Level II media. Unlike ketoconazole, which is not poorly soluble at pH values of about 3–4, danazol is a poorly soluble compound, and even small changes in the interaction between pH buffering species and proteins could impact the solubilization capacity of proteins.
2. equilibrium solubility data in Level III FeSSGF-V3 was slightly more variable. Variability in the vesicular/micellar structures in the presence of proteins could contribute to the variability of solubility of this poorly soluble compound.

## 4 Discussion

Drug apparent equilibrium solubility is a key parameter for the estimation of drug dissolution characteristics after oral administration. The usefulness of an in vitro methodology in estimating drug apparent solubility in the luminal contents should be evaluated against luminal data, which, to date, could be based on the drug equilibrium solubility in luminal contents measured ex vivo. It is extremely difficult to avoid one freeze–thaw cycle when aiming at equilibrium solubility measurements in aspirates from the gastrointestinal lumen. Based on data collected in this study, the impact of one freeze–thaw cycle on the pH of antral aspirates collected after a high-calorie, high-fat meal does not seem to be substantial in relation to pH and buffer capacity; only the buffer capacity of the HGF_fed_^135^ pooled sample seems to have deviated from the average values estimated immediately upon aspirations (Table 1). Therefore, compared with the fasted state,^26^ the antral aspirates collected after a high-calorie, high-fat meal are less sensitive to buffer capacity changes due to one freeze–thaw cycle. However, the type and number of colloidal species in the stomach and the impact of one freeze–thaw cycle are not known. To date, only data after freezing of intestinal aspirates collected after a liquid meal have been reported.^27^ The potential impact of one freeze–thaw cycle on the colloidal species in the antrum and on the apparent solubility data collected in this investigation should be acknowledged.

A point estimate for the apparent drug equilibrium solubility in antral contents after a high-calorie, high-fat meal and a glass of water with the drug 30 min after initiation of meal administration to healthy young adults^15,16^ cannot give full insight into the solubility as a function of time after water administration in different individuals as both intersubject and intrasubject variability in pH and colloidal species concentrations is high, e.g., refs (9,14). Variability aspects could be addressed by performing apparent equilibrium solubility measurements based on the design-of-experiment approach, covering relevant intervals for each of the selected factors and subsequent mathematical modeling, as it has been suggested for the estimation of equilibrium solubility in the fasted upper small intestine,^28^ by also addressing the impact of time after water (and drug) administration. Based on the experience, to date, such an approach would be unlikely to be widely accepted by the pharmaceutical industry. Although this realization limits the expectations of *in silico* physiologically based biopharmaceutics (PBB) modeling approaches, a useful step toward improving the efficiency of *in silico* PBB modeling methodologies in the prediction of food effects would be the estimation of a reliable point estimate for intragastric apparent drug solubility in the fed state.^2^

Equilibrium solubility of ketoconazole and danazol in Level I and Level II FeSSGF-V2 is much lower than the corresponding point estimates for the apparent equilibrium solubility in antral contents in the fed state. Also, FeSSGF-V2 can be associated with two potential limitations:

1. Buffered solutions with the same pH could show similar resistance in changing the pH toward one direction but different resistance in changing the pH toward the other direction [e.g., ref (29)]. In FeSSGF-V2, buffer capacity (the resistance to change the pH) reflects antral buffer capacity only toward more acidic values (Section 2.4.1). For ionizable compounds, depending on the solubility, buffer capacity of the medium might be exceeded and significantly shift the pH at equilibrium. For poorly soluble compounds, this is more frequently observed when the equilibrium solubility of a salt is measured, especially when the amount of solid in excess is not monitored [e.g., ref (25)]. Therefore, for universal application, biorelevant media employed in solubility measurements should have buffer capacity similar to that of gastric aspirates in both pH directions.
2. Lipofundin in Level II FeSSGF-V2 is difficult to access, at least in some countries. Therefore, for universal application, it would be preferred to simulate the presence of lipids by using another easily accessible, commercially available product, e.g., FEDGASgel, that could serve this purpose.

Due to the easy access of materials and the preparation method, FEDGAS has recently gained popularity in achieving Level II simulation of the environment in stomach after a high-calorie, high-fat meal and a glass of water 30 min after initiation of meal administration. Relevant solubility data have been implemented in various physiologically based biopharmaceutics (*in silico*) modeling approaches.^30−33^ One issue is that solubility data collected in FEDGAS pH = 6.0 or in FEDGAS pH = 4.5 may not be useful as the specific pH values are unlikely to exist in stomach after a high-calorie, high-fat meal and a glass of water 30 min after initiation of meal administration, at least at times later than first half-hour after water administration (e.g., ref (9) and references therein). Another issue is that, based on data collected in the present investigation, apparent equilibrium solubility of ketoconazole or danazol in FEDGAS pH = 3 (Level II simulation) is two to three times higher than the corresponding point estimates for solubility in antral contents (Figure 2).

In the present investigation, a new biorelevant medium, FeSSGF-V3 is proposed.

FeSSGF-V3 lacks the potential limitations of FeSSGF-V2 mentioned above. Also, with FeSSGF-V3, the Level III biorelevance^5^ of gastric contents after a high-calorie, high-fat meal and a glass of water 30 min after initiation of meal administration is proposed for the first time, by simulating both the presence of proteins and the apparent viscosity in antral contents by using an easily accessible, commercially available product (Régilait skimmed milk powder). Apparent viscosity could be important if FeSSGF-V3 is also considered for biorelevant in vitro disintegration and dissolution studies.^34^

Level III FeSSGF-V3 prepared by using hydrochloric acid, commercially available FEDGASgel [16.6% of the amount of FEDGASgel that is currently recommended (biorelevant.com)], and Régilait skimmed milk powder provides a basis for simulating the pH, buffer capacity, osmolality, lipid content, and protein content in the antrum during the first 3 h after a high-calorie, high-fat meal and a glass of water to healthy adults. Equilibrium solubility of ketoconazole and danazol in Level III FeSSGF-V3 prepared with hydrochloric acid is comparatively closest to the point of the corresponding estimates for equilibrium solubility in antral contents in the fed state; data are 24 and 12% higher than the estimated point solubility for ketoconazole and danazol, respectively.

## 5 Conclusions

Based on data from this study, Level III FeSSGF-V3 prepared by using HCl as the principal pH controlling species is useful for point estimating ketoconazole and danazol solubility in antral contents after a high-calorie, high-fat meal and a glass of water to healthy adults. Performance of additional carefully performed intubation studies so that they reflect as much as possible the actual luminal conditions in BA/BE studies and confirmation of the usefulness of Level III FeSSGF-V3_HCl_ using other model active pharmaceutical ingredients would further increase the reliability of *in silico* PBB modeling or biorelevant *in vitro* modeling approaches in the prediction of food effects on oral drug absorption.

## Supporting Information Available

Details on the The Supporting Information is available free of charge at https://pubs.acs.org/doi/10.1021/acs.molpharmaceut.4c01038.

Variability of physicochemical characteristics of pooled gastric aspirates, method of preparation of Level I/Level II/Level III FeSSGF-V3 media, within-day and between-day variability and the impact of storage conditions on the physicochemical characteristics of Level III FeSSGF-V3 media, equilibration times in solubility measurements in Level III FeSSGF-V3HCl and HGFfed45, and adsorption of ketoconazole and danazol on filters (PDF)
