# Supplementary material for: Simulation of Antral Conditions for Estimating Drug Apparent Equilibrium Solubility after a High-Calorie, High-Fat Meal
Source: Mol Pharm. 2025 Jan 15;22(2):871–81. doi: 10.1021/acs.molpharmaceut.4c01038 (PMC11795529; doi:10.1021/acs.molpharmaceut.4c01038)
Supplement: Supplementary file 1 — mp4c01038_si_001.pdf [file mp4c01038_si_001.pdf]

**Simulation of antral conditions for estimating drug apparent equilibrium solubility after a high-calorie, high-fat meal**

Christos Reppas, Christina Chorianopoulou, Ioanna Karkaletsi, Shirin Dietrich, Andriani Bakolia, Maria Vertzoni*

Department of Pharmacy, National and Kapodistrian University of Athens, Zografou, Greece

# SUPPLEMENTARY INFORMATION

**SI1.** Variability of physicochemical characteristics of pooled gastric aspirates ........ 2

**SI2.** Method of preparation of Level I/Level II/Level III FeSSGF-V3 media ........ 3

**SI3.** Within-day and between-days variability and the impact storage conditions on the physicochemical characteristics of Level III FeSSGF-V3 media ........ 6

**SI4.** Equilibration times in solubility measurements in Level III FeSSGF-V3$_{HCl}$ and $HGFfed45$ ........ 9

**SI5.** Adsorption of ketoconazole and danazol on filters ........ 10

# SI1.Variability of physicochemical characteristics of pooled gastric aspirates

**Table SI1.1** Variability of pH, buffer capacity, and osmolality measurements (3 replications per measurement) in pooled samples generated after one freeze-thaw cycle of aspirates collected by Pentafragka et al. 2020a and 2020b.

| | | $HGF_{fed}^{15}$ | | | $HGF_{fed}^{195}$ | | |
|---|---|---|---|---|---|---|---|
| | | #1 | #2 | #3 | #1 | #2 | #3 |
| **pH** | | 3.85 | 3.86 | 3.87 | 1.97 | 2.01 | 2.02 |
| **Buffer capacity HCl [(mmol/L)/ΔpH]** | **Titration with HCl** | 11.4 | 11.2 | 10.8 | Not measured | | |
| | **Titration with NaOH** | 8.2 | 7.6 | 7.4 | 22.0 | 22.5 | 22.5 |
| **Osmolality (mOsmol/kg)** | | 154 | 154 | 158 | 277 | 271 | 263 |


Pentafragka C., Vertzoni M., Dressman J., Symillides M., Goumas C., Reppas C. Characteristics of contents in the upper gastrointestinal lumen after a standard high-calorie high-fat meal and implications for the in vitro drug product performance testing conditions. *Eur. J. Pharm. Sci.* **155**:105535 (2020a).

Pentafragka C., Vertzoni M., Symillides M., Goumas K., Reppas C. Disposition of two highly permeable drugs in the upper gastrointestinal lumen of healthy adults after a standard high-calorie, high-fat meal. *Eur. J. Pharm. Sci.* **149**:105351 (2020b).

# SI2. Method of preparation of Level I/Level II/Level III FeSSGF-V3 media

## FeSSGF-V3$_{HCl}$

### Level I FeSSGF-V3$_{HCl}$

For the preparation of 100 mL of Level I FeSSGF-V3$_{HCl}$ :

- ~70 mL of deionized water is added into a 100-mL volumetric flask
- **1.2 mL of 0.1 M HCl** is added to the flask
- deionized water is added up to the mark (pH is adjusted to 3.0, if it is necessary, before reaching the mark)

### Level II FeSSGF-V3$_{HCl}$

For the preparation of 100 mL of Level II FeSSGF-V3$_{HCl}$:

- ~70 mL of deionized water is added into a beaker of 100mL
- **0.7 mL of 0.1 M HCl** is added to the beaker adjusting the pH to 3.0
- **0.802 g of sodium chloride** is dissolved to the solution
- **2.83 g of FEDGAS gel** is weighed into a beaker of 50 mL
- part of the solution is gradually transferred to the small beaker and stirred until the gel is completely dissolved
- pH is adjusted to exactly pH 3.0 using **1.3 mL of HCl 0.1 M**
- the rest of the HCl/NaCl solution and the solution with gel are quantitatively transferred to a 100-mL volumetric flask
- deionized water is added up to the mark (pH is adjusted to 3.0, if it is necessary, before reaching the mark)

### Level III FeSSGF-V3$_{HCl}$

For the preparation of 100 mL of Level III FeSSGF-V3$_{HCl}$:

- ~80 mL of deionized water is added into a beaker of 100mL
- **6.5 mL of 1 M HCl** is added to the beaker (pH 1.29)
- **2.83 g of FEDGAS gel** is weighed into a beaker of 50 mL
- part of solution is gradually transferred to the small beaker and stirred until the gel is completely dissolved
- **6.1 g of Régilait® powder** is weighed into a beaker of 250 mL
- the rest of HCl/NaCl solution and the solution with the gel is quantitatively transferred to the 250-mL beaker and stirred until homogenization
- the suspension is quantitatively transferred to a 100-mL volumetric flask
- deionized water is added up to the mark

# FeSSGF-V3$_{Acetates}$

## Level I FeSSGF-V3$_{Acetates}$

For the preparation of 100 mL of Level I FeSSGF-V3$_{Acetates}$:

- ~80 mL of deionized water is added into a beaker of 100 mL
- **0.771 mL of glacial acetic acid** is added to the beaker (pH 2.86)
- the pH is adjusted to 3.0 using **0.4 mL of NaOH 0.5 M**
- the solution is quantitatively transferred to a 100-mL volumetric flask
- deionized water is added up to the mark

## Level II FeSSGF-V3$_{Acetates}$

For the preparation of 100 mL of Level II FeSSGF-V3$_{Acetates}$ :

- ~60 mL of deionized water is added into a beaker of 100 mL
- **0.771 mL of glacial acetic acid** is added to the beaker (pH 2.72)
- **0.327 g of sodium chloride** is dissolved in the solution
- the pH is adjusted to 3.0 using **0.7 mL of NaOH 0.5 M**
- **2.83 g of FEDGAS gel** is weighed into a beaker of 50 mL
- part of the solution is gradually transferred to the s,all beaker and stirred until the gel is completely dissolved (pH 3.14)
- pH is to 3.0 using **0.15 mL of HCl 1M**
- the rest of the buffer and the solution with gel are quantitatively transferred to a 100-mL volumetric flask
- deionized water is added up to the mark

## Level III FeSSGF-V3$_{Acetates}$

For the preparation of 100 mL of Level III FeSSGF-V3$_{Acetates}$ :

- ~60 mL of deionized water is added into a beaker of 100 mL
- **0.386 mL of concentrated acetic acid** is added to the beaker (pH 2.91)
- the pH is adjusted to 3.0 using **0.15 mL of NaOH 0.5 M**
- **2.83 g of FEDGAS gel** is weighed into a beaker of 50 mL
- part of the solution is gradually transferred to the small beaker and stirred until the gel is completely dissolved
- **6.1 g of Régilait powder** is weighed into a beaker of 250 mL
- The rest of the buffer and the solution with the gel are quantitatively transferred to the 250-mL beaker and stirred until homogenization (pH 4.64)
- pH is adjusted to 3.0 using **6.6 mL of HCl 1 M**
- the suspension is quantitatively transferred to a 100-mL volumetric flask
- deionized water is added up to the mark

## FeSSGF-V3$_{FEDGAS}$

### Level I FeSSGF-V3$_{FEDGAS}$

For the preparation of 100 mL of Level I FeSSGF-V3$_{FEDGAS}$:

- ~80 mL of deionized water is added into a beaker of 100 mL
- **4.08 mL of FEDGAS buffer** is added to the beaker (pH 2.57)
- the pH is adjusted to 3.0 using **1.7 mL of NaOH 0.5 M**
- the solution is quantitatively transferred to a 100-mL volumetric flask
- deionized water is added up to the mark

### Level II FeSSGF-V3$_{FEDGAS}$

For the preparation of 100 mL of Level II FeSSGF-V3$_{FEDGAS}$ :

- ~70 mL of deionized water is added into a beaker of 100 mL
- **4.08 mL of FEDGAS buffer** is added to the beaker (pH 2.67)
- **0.482 g of sodium chloride** is dissolved in the solution
- the pH is adjusted to 3.0 using **1.5 mL of NaOH 0.5 M**
- **2.83 g of FEDGAS gel** is weighed into a beaker of 50 mL
- part of the solution is gradually transferred to the small beaker and stirred until the gel is completely dissolved (pH 3.07)
- pH is adjusted to 3.0 using **0.1 mL of HCl 1 M**
- the rest of the buffer and the solution with gel are quantitatively transferred to a 100-mL volumetric flask
- deionized water is added up to the mark

### Level III FeSSGF-V3$_{FEDGAS}$

For the preparation of 100 mL of Level III FeSSGF-V3$_{FEDGAS}$ :

- ~70 mL of deionized water is added into a beaker of 100 mL
- **1 mL of FEDGAS buffer** is added to the beaker (pH 2.88)
- the pH is adjusted to 3.0 using **0.30 mL of NaOH 0.5 M**
- **2.83 g of FEDGAS gel** is weighed into a beaker of 50 mL
- part of the solution is gradually transferred to the small beaker and stirred until the gel is completely dissolved
- **6.1 g of Régilait powder** is weighed into a beaker of 250 mL
- the rest of the buffer and the solution with the gel are quantitatively transferred to the 250-mL beaker and stirred until homogenization (pH 5.46)
- pH is adjusted to 3.0 using **6.4 mL of HCl 1 M**
- the suspension is quantitatively transferred to a 100-mL volumetric flask
- deionized water is added up to the mark

## SI3. Within-day and between-days variability and the impact storage conditions on the physicochemical characteristics of Level III FeSSGF-V3 media

**Table SI3.1:** Within-day and between-days variability in the physicochemical characteristics of Level III $\mathrm{FeSSGF-V3_{HCl}}$, Level III $\mathrm{FeSSGF-V3_{acetates}}$ and Level III $\mathrm{FeSSGF-V3_{FEDGAS}}$.

| Variability | Medium | pH | Buffer capacity measured with NaOH 0.1 M (mmol/L)/ΔpH | Buffer capacity measured with HCl 0.1 M (mmol/L)/ΔpH | Osmolality (mOsm/kg) | Apparent viscosity at $100s^{-1}$ and 37°C (mPa·s) |
|---|---|---|---|---|---|---|
| Within-Day[1] | Level III $\mathbf{FeSSGF-V3_{HCl}}$ | 2.99 (0.01) | 15.31 (0.10) | 24.03 (0.29) | 288.00 (0.67) | 156 (69) |
| | Level III $\mathbf{FeSSGF-V3_{acetates}}$ | 3.00 (0.00) | 26.75 (0.74) | 27.78 (0.35) | 365.1 (3.1) | 263.56 (0.72) |
| | Level III $\mathbf{FeSSGF-V3_{FEDGAS}}$ | 3.04 (0.01) | 22.72 (0.25) | 30.56 (0.10) | 302.4 (9.4) | 215 (39) |
| Between-Days[2] | Level III $\mathbf{FeSSGF-V3_{HCl}}$ | 3.00 (0.01) | 15.38 (0.11) | 24.51 (0.49) | 308 (29) | 208 (52) |
| | Level III $\mathbf{FeSSGF-V3_{acetates}}$ | 3.01 (0.01) | 26.69 (0.67) | 27.31 (0.85) | 366.7 (2.3) | 346 (78) |
| | Level III $\mathbf{FeSSGF-V3_{FEDGAS}}$ | 3.01 (0.02) | 22.99 (0.67) | 29.6 (1.1) | 306.2 (5.3) | 279 (93) |

[1]Mean(SD) (n=3 preparations of the medium on one day)

[2]Mean(SD) (n=3 different days. 3 preparations of the medium were performed within the same day)

**Table SI3.2**: The impact of storage and temperature on the physicochemical characteristics of Level III $\mathrm{FeSSGF}-\mathrm{V3}_{\mathrm{HCl}}$, Level III $\mathrm{FeSSGF}-\mathrm{V3}_{\mathrm{acetates}}$ and Level III $\mathrm{FeSSGF}-\mathrm{V3}_{\mathrm{FEDGAS}}$[1].

| Medium | Impact of storage and temperature | pH | Buffer capacity measured with NaOH 0.1 M (mmol/L)/ΔpH | Buffer capacity measured with HCl 0.1 M (mmol/L)/ΔpH | Viscosity at 100 $s^{-1}$ and 37°C (mPa·s) |
|---|---|---|---|---|---|
| **Level III $\mathbf{FeSSGF-V3_{HCl}}$** | After 1h at room temperature | 3.00 (0.01) | 25.00 (0.50) | 15.50 (0.50) | 261 (52) |
| | After 2h in shaking bath at 37 °C | N/A | N/A | N/A | 218 (36) |
| | After 4h in shaking bath at 37 °C | N/A | N/A | N/A | 198 (34) |
| | After 24 in shaking bath at 37 °C | 3.01 (0.01) | 25.17 (0.58) | 17.50 (0.50) | 178 (58) |
| **Level III $\mathbf{FeSSGF-V3_{acetates}}$** | After 1h at room temperature | 3.02 (0.00) | 26.00 (0.00) | 26.33 (0.29) | 354 (89) |
| | After 2h in shaking bath at 37 °C | N/A | N/A | N/A | 296 (72) |
| | After 4h in shaking bath at 37 °C | N/A | N/A | N/A | 291 (61) |
| | After 24 in shaking bath at 37 °C | 3.03 (0.01) | 27.00 (1.00) | 26.00 (0.50) | 241 (54) |
| **Level III $\mathbf{FeSSGF-V3_{FEDGAS}}$** | After 1h at room temperature | 3.01 (0.01) | 22.50 (0.00) | 29.83 (0.29) | 238 (23) |
| | After 2h in shaking bath at 37 °C | N/A | N/A | N/A | 223.5 (8.2) |
| | After 4h in shaking bath at 37 °C | N/A | N/A | N/A | 216 (19) |
| | After 24 in shaking bath at 37 °C | 3.01 (0.00) | 23.17 (0.29) | 29.50 (0.00) | 201 (29) |

[1]NM=not measured; Mean(SD) (n=3 difference samples from a medium prepared once)

## SI4. Equilibration times in solubility measurements in Level III FeSSGF-V3$_{HCl}$ and $HGF_{fed}^{45}$

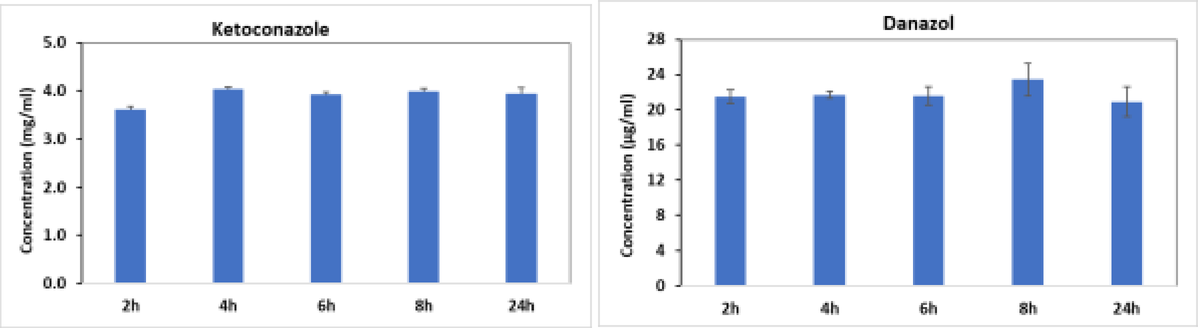


**Figure SI4.1** Preliminary equilibrium solubility experiments of ketoconazole and danazol in Level III FeSSGF-V3$_{HCl}$

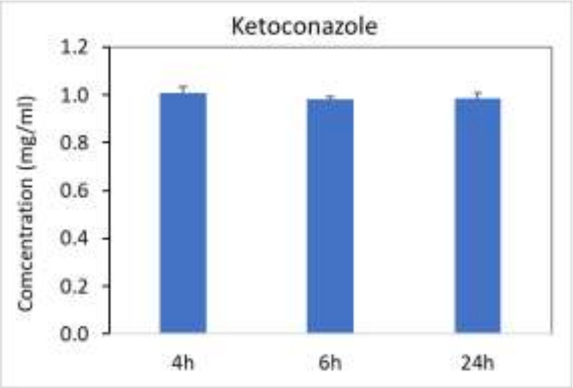


**Figure SI4.2** Preliminary equilibrium solubility experiments of ketoconazole in a $HGF_{fed}^{15}$ sample created from aspirates collected in the study of Pentafragka et al. (2020a)

# SI5. Adsorption of ketoconazole and danazol on filters

**Table SI5.1** Equilibrium solubility measurements of ketoconazole in pH 3.5, pH 4.0 and pH 5.0. Three different samples were used.

| | | Ketoconazole Concentration (mg/ml) | | | |
|---|---|---|---|---|---|
| | **Filtration** | **Mean** | **SD** | **% RSD** | **% adsorbed** |
| **pH 3.5** | **1st** | 2.8763 | 0.0841 | 2.92 | 1.63 |
| | **2nd** | 2.8250 | 0.2050 | 7.26 | |
| **pH 4.0** | **1st** | 0.9297 | 0.0027 | 0.29 | 0.23 |
| | **2nd** | 0.9288 | 0.0106 | 1.14 | |
| **pH 5.0** | **1st** | 0.0825 | 0.0058 | 7.06 | 6.16 |
| | **2nd** | 0.0775 | 0.0020 | 2.56 | |

**Table SI5.2** Equilibrium solubility measurements of danazol in Level II FeSSIF. Three different samples were used.

| | | Danazol | |
|---|---|---|---|
| **Sample #** | **Filtration** | **Concentration (ug/ml)** | **% adsorbed** |
| **1** | **1st** | 26.61 | 1.04 |
| | **2nd** | 26.34 | |
| **2** | **1st** | 26.22 | 3.18 |
| | **2nd** | 25.39 | |
| **3** | **1st** | 26.44 | 3.78 |
| | **2nd** | 25.44 | |
